# The association between basic pension insurance and fertility intentions among flexibly employed workers in China’s new forms of employment

**DOI:** 10.3389/fpubh.2026.1892526

**Published:** 2026-08-26

**Authors:** Qizhong Zheng, Lulin Zhou

**Affiliations:** School of Management, Jiangsu University, Zhenjiang, China

**Keywords:** basic pension insurance, China, fertility intention, flexible workers, new forms of employment

## Abstract

**Introduction:**

Following the relaxation of China’s fertility policy, the nation-a rapidly growing group in the platform economy-are predominantly young, of reproductive age, and generally lack adequate social security coverage. Their fertility intentions therefore merit particular attention.

**Methods:**

We conducted a stratified purposive survey across 23 provinces and municipalities, collecting 604 valid responses from NFE flexible workers. The analytical sample was restricted to ever‑married respondents with at least one child (*N* = 336). Using Logit, Probit, Poisson, and OLS regression models, complemented by Propensity Score Matching (PSM), we estimated the association between participation in basic pension insurance and two measures of fertility intention: willingness to have a second child (Plan1) and ideal number of children (Plan2).

**Results:**

Participation in basic pension insurance is significantly negatively associated with both Plan1 and Plan2 across all models. The average marginal effect from the Logit model indicates a reduction of 8.5 percentage points in the probability of intending a second child (*p* < 0.01). The PSM‑estimated average treatment effect on the treated (ATT) shows that participants have, on average, about 23.5 percentage points lower second‑child intention than non-participants. Heterogeneity analyses reveal that the negative association is stronger for non-agricultural hukou holders and for those who engage in NFE as their sole or primary job, while it is weaker for secondary-job holders.

**Discussion:**

These findings suggest that basic pension insurance may substitute for the traditional “raising children for old‑age support”motive, thereby dampening fertility intentions among NFE flexible workers. To balance old-age protection with fertility promotion, policy interventions could consider adjusting contribution rates, expanding social security coverage, and strengthening employment protection for female workers. Further research using panel or quasi‑experimental designs is needed to establish causality.

## 1 Introduction

Since the dawn of the 21st century, China’s demographic landscape has undergone a profound transformation, marked by an accelerating aging process and a persistent decline in birth rates. Data from the Seventh National Population Census in 2021 revealed that the total fertility rate (TFR) has plummeted to 1.3, falling significantly below the population replacement threshold. This downward trajectory was further evidenced in 2022, when the national population experienced an absolute contraction of 850,000. Despite the transition to a ‘three-child policy,’ fertility intentions remain subdued, highlighting the limited impact of recent policy shifts on reversing demographic trends (1).

Driven by the rapid advancement of digital infrastructure and the expansion of mobile terminal applications, new forms of employment based on the platform and sharing economies have grown rapidly. The term “new forms of employment” has entered public discourse, and the number of people engaged in such work is steadily increasing (2). According to the Ninth National Survey on the Situation of the Chinese Workforce, there are currently 84 million workers in new forms of employment nationwide. This group predominantly consists of young adults of reproductive age, making them a crucial demographic for unleashing China’s fertility potential. However, this group commonly suffers from a deficit in adequate social security protections.

Basic pension insurance is a cornerstone of China’s social security system, designed to provide a basic standard of living for the older population and ensure they are cared for in their later years. While contributing to social stability and security, it has also challenged the traditional cultural norm of “raising children for old-age security,” thereby influencing fertility intentions (3). Although basic pension insurance system theoretically covers NFE flexible workers, the specific nature of their work environment and relatively high contribution rates have led to some being effectively “crowded out” of the pension system (4).

Given this context, this paper employs Poisson, OLS, Probit, and Logit models to examine the statistical association between basic pension insurance and the fertility intentions of NFE flexible workers. The aim is to provide policy recommendations for improving the social security system for this group, and to inform policy discussions that may support fertility intentions in the context of the national fertility rate.

Unlike regular employees in standard employment relationships, flexibly employed workers in NFE are characterized by precarious work arrangements, volatile income streams, and limited access to employer-sponsored social benefits. These features make the trade-off between pension insurance participation and fertility decisions particularly salient for this group. First, the opportunity cost of pension contributions—foregone current income that could otherwise support child-rearing—is more acutely felt by workers with irregular earnings. Second, the substitution effect may operate more strongly among NFE workers, as their limited institutional coverage heightens the perceived role of pension insurance as a substitute for family-based old-age support, while simultaneously amplifying income-related constraints on fertility decisions. Third, the absence of employer-provided maternity benefits and job security further complicates the fertility calculus for this population. Thus, examining the pension-fertility nexus among NFE flexible workers not only fills an empirical gap but also extends the theoretical application of the substitution and income effects to a context characterized by institutional precarity and economic vulnerability.

## 2 Literature review

### 2.1 Current state and multifaceted influences on fertility intentions

In recent years, the declining fertility intentions among China’s reproductive-age population have become a central focus of demographic research. This trend is corroborated by multiple large-scale surveys. For instance, a study by Zhang et al. (5) involving 2,316 women of childbearing age in Shanxi Province found that only 31.00% expressed a clear intention to have children, indicating a relatively low level. Liu and Lu (2025), analyzing data from the Chinese General Social Survey (CGSS) since 2010, also found that both childless individuals and those with children desired an average of about 1.5 children, with extremely low intentions for a second or third child. This suggests China has generally entered an era of low fertility intentions (6). This phenomenon is not unique to China. In South Korea, which also faces ultra-low fertility rates, a systematic review by Kim and Yi (2024) found that women’s fertility intentions were positively influenced by factors such as spousal involvement in childcare, their own education level, and socioeconomic status, while age, parenting stress, and a disproportionate share of housework significantly weakened their intentions (7).

Scholars have explored the complex factors influencing fertility intentions from multiple perspectives. At the macro level, the study by Zhai et al. (2025) is representative, highlighting the unique nature of China’s “anticipatory” fertility decline. They argue that the core constraints are the “care dilemma,” “education anxiety,” and “housing burden.” These pressures, exacerbated by the weakening of family support functions and intensifying social competition, significantly increase the economic and psychological costs of childbearing, raising, and educating children (8). Liang et al. (2025), using grounded theory, constructed a model of factors influencing fertility intentions, incorporating policy, social, family, and individual dimensions into their analytical framework, emphasizing the importance of multi-stakeholder collaboration (9). At the micro level, individual characteristics and health status cannot be ignored. The study by Zhang et al. (2025) particularly emphasizes the role of health, finding that a history of miscarriage, medical or gynecological conditions, and especially pelvic floor disorders like postpartum urinary incontinence and uterine prolapse, significantly reduce women’s willingness to have another child (5). Furthermore, socio-psychological factors such as employment stability (Liu & Lu, 2025), residential mobility (Xiao et al., 2023), and levels of feminist identification (Qiao et al., 2024) have also been confirmed to be significantly associated with fertility intentions (6, 10, 11).

However, existing research often approaches fertility intentions from static or unidimensional perspectives, leaving the complexity of the issue underexplored. For example, most quantitative studies rely on cross-sectional data, making it difficult to capture the dynamic evolution of individual fertility intentions over the life course and in response to changing external environments Grzenda (12). Furthermore, while some studies have begun to address the gap between “fertility intention” and “fertility behavior” (Spéder & Bálint, 2024; Grzenda, 2024), the mediating mechanisms and psychological pathways involved remain insufficiently explored. Research focusing on specific vulnerable or emerging employment groups is particularly scarce (12, 13).

### 2.2 Theoretical debates on the relationship between basic pension insurance and fertility intentions

Basic pension insurance, a core component of the social security system, has a contentious relationship with individual fertility decisions, with academic debate centered on two main theoretical perspectives: the “substitution effect” and the “income effect” (14). Some scholars advocate for the “substitution effect,” arguing that pension insurance, as a socialized old-age support mechanism, weakens the traditional motive of “raising children for old age,” thereby crowding out fertility intentions. Using CFPS 2018 data, Wang (2023) found that participation in pension insurance reduced individuals’ fertility intentions by 2.8%, with a more pronounced effect among men and rural residents (15). Yu and Ming (2022), in their study on farmers’ intentions to have a third child, also confirmed a significant “crowding out” effect of pension insurance (16). Research by Jing et al. (2022) and Xiong and Zhang (2022), focusing on flexible workers, similarly found that social pension insurance generally has a crowding-out effect on fertility intentions. Although the “New Rural Social Pension Insurance” showed a positive impact under specific conditions, this positive effect weakened when the concept of “raising children for old age” was introduced as a moderating variable. This suggests that traditional norms remain deeply ingrained and that social pensions have not yet fully replaced the family’s role in old-age support (3, 17). Xie and Ma (2023) even found that urban residents not covered by basic pension insurance were more inclined to have children, providing indirect support for the existence of a substitution effect (18).

Conversely, another group of scholars emphasizes the “income effect.” Fan et al. (2025), utilizing CLDS data and individual fixed-effects models, found that basic pension insurance significantly increased the fertility intentions of married young adults, particularly among those aged 36–45, males, rural residents, and low-income groups. The proposed mechanism is that pension insurance enhances life satisfaction and current consumption, thereby promoting fertility intentions, manifesting as a significant “income effect” (19). Li (2022), in a study on the New Rural Social Pension Insurance, reached a similar conclusion, finding that it significantly boosted the fertility intentions of rural residents, with the strongest effect observed in middle-income families (20). Furthermore, Lin (2022), based on CGSS 2017 data, found that participating in pension insurance had a significant positive impact on women’s preference for having a daughter, offering a gender-specific perspective on the differential effects of pension insurance (21).

The divergence in these findings highlights the complexity of the relationship between old-age insurance and fertility intentions. These contradictions may stem from differences in study samples (e.g., urban vs. rural, age groups), methodologies (cross-sectional vs. panel data), definitions of core variables (dimensions for measuring fertility intention), and types of pension schemes (e.g., urban employee, urban–rural resident, new rural). While existing research has begun to explore heterogeneity (Fan et al., 2025; Jing et al., 2022) (17, 20), there is a lack of deeper theoretical integration and empirical testing regarding the coexistence of “income” and “substitution” effects and the conditions under which one effect dominates. For instance, an individual’s life cycle stage, income level, and trust in the social pension system could be key moderating variables determining which effect prevails.

While the substitution and income effects have been extensively discussed in general population studies, their applicability to NFE flexible workers warrants particular theoretical attention. The conventional framework assumes relatively stable employment and predictable income—conditions that do not hold for NFE workers. For this group, the substitution effect may be amplified by the absence of alternative old-age support mechanisms, while the income effect may be dampened by the immediate liquidity constraints imposed by irregular earnings. Moreover, the interaction between employment instability and pension participation creates a distinct behavioral dynamic: workers facing income volatility may prioritize short-term consumption (including child-rearing) over long-term insurance, yet the prospect of inadequate old-age security may simultaneously motivate pension enrollment. This dual tension—between immediate economic pressures and long-term security needs—constitutes the core theoretical puzzle that this study addresses.

### 2.3 Progress and limitations in research on flexibly employed workers in new forms of employment

With the rapid growth of the platform and sharing economies, the number of flexibly employed workers in new forms of employment has expanded swiftly. Their characteristics—youth, ambiguous labor relations, and inadequate social security—make them a critical focus for both unleashing fertility potential and strengthening social safety nets (Jing et al., 2022; Xiong & Zhang, 2022) (3, 17). Current research has begun to explore the interaction between old-age security and fertility intentions within this specific group. Jing et al. (2022) found that social pension insurance generally has a crowding-out effect on the intention to have a second child among these workers, although the impact varies by scheme type, with the “New Rural Social Pension Insurance” showing a relatively more positive influence (15). Xiong and Zhang (2022) further revealed the complexity of this interaction, noting that the “raising children for old age” norm still dominates fertility decisions for flexible workers, and social pensions have not yet effectively substituted for family support. In fact, obtaining pension coverage sometimes lowered their willingness to have more children (3). These studies provide an initial sketch of the trade-off between old-age expectations and fertility decisions faced by this group.

However, research specifically targeting NFE flexible workers is still in its infancy and has notable limitations. First, the number of studies is extremely limited, with only a few scholars conducting targeted research (Huang et al., 2023; Cheung et al., 2024) (22, 23). Moreover, many rely on data from CGSS or CLDS collected in 2017 or 2018, periods before the full flourishing of the new forms of employment and the implementation of the “three-child policy.” Consequently, their explanatory power and timeliness regarding the current situation may be insufficient (24). Second, existing studies often treat flexible workers as a relatively homogeneous group. In reality, this group is internally complex, encompassing various occupations such as food delivery riders, ride-hailing drivers, platform streamers, and freelancers, who differ greatly in income, work intensity, career prospects, and social security participation patterns. These heterogeneities and their impact on fertility intentions have not been examined in detail. Third, research largely focuses on the direct effect of pension insurance participation, with relatively superficial analysis of the influencing pathways (25). For example, there is a lack of in-depth exploration of indirect effects on fertility decisions stemming from income volatility expectations due to job instability, occupational injury risks, and the absence of employer-provided maternity-related benefits (e.g., maternity leave, parental leave). International experiences, such as the survey experiment by Wang et al. (2024) in Singapore on the impact of flexible work arrangements on fertility intentions, offer valuable insights into how improving working conditions could enhance fertility intentions, particularly for women. However, similar research within China remains absent (14). Therefore, there is an urgent need to use more recent, representative data combined with qualitative methods to delve deeper into the real fertility dilemmas and needs of this large and growing population. This would provide a solid academic foundation for formulating precise and effective fertility support policies.

## 3 Research design

### 3.1 Data collection

Given the absence of suitable variables in existing social statistics to measure the association between basic pension insurance and the fertility intentions of NFE flexible workers, this study employed a stratified purposive sampling strategy. Samples were drawn from 23 provinces and municipalities, including Jiangsu, Shandong, and Hunan, for a field questionnaire survey. A total of 650 questionnaires were distributed, and 604 valid responses were collected, yielding a response rate of 92.6%. After removing cases with missing values, the composition of the 604 respondents is as follows: 514 males and 88 females; 414 with agricultural household registration (hukou) and 188 with non-agricultural hukou; 266 unmarried, 282 married, 52 divorced, and 2 widowed.

The high proportion of agricultural hukou holders in our analytical sample (approximately 67%) reflects the broader demographic composition of China’s NFE workforce. According to the Ninth National Survey on the Situation of the Chinese Workforce conducted by the All-China Federation of Trade Unions, NFE workers—primarily comprising truck drivers, ride-hailing drivers, delivery riders, and food delivery couriers—are predominantly young and middle-aged males with a notably high proportion of agricultural hukou holders. Specific occupation-level data further corroborate this pattern: survey data from the Chinese Academy of Social Sciences indicate that approximately 70% of food delivery riders hold agricultural hukou, while industry reports suggest that over 70% of delivery couriers also originate from rural areas. Thus, the agricultural hukou composition of our sample—67%—is broadly consistent with the national demographic profile of NFE workers, supporting the representativeness of our analytical sample for this population.

The decision to restrict the analytical sample to ever-married respondents with at least one child was guided by both theoretical and methodological considerations. Theoretically, the decision to have a second child is fundamentally different from the decision to have a first child: the former involves considerations of resource allocation across existing children, the potential disruption to established family routines, and the opportunity costs of additional childbearing—considerations that are simply not applicable to childless individuals. Methodologically, including childless or unmarried respondents in an analysis of second-child intentions would introduce measurement invalidity, as the dependent variable would lack substantive meaning for a substantial portion of the sample. The original full sample of 604 respondents included 266 unmarried individuals, among whom a considerable proportion had no children; excluding them does not constitute selection on the dependent variable but rather ensures that the analytical sample aligns with the conceptual definition of the outcome of interest. However, we acknowledge that this restriction limits the generalizability of our findings to the broader NFE workforce, particularly to unmarried or childless NFE workers whose fertility decisions may follow different logics. We discuss this limitation in Section 6.

Of the 604 respondents who completed the survey, we restricted the analytical sample to those who were ever-married (including married, divorced, and widowed) and had at least one child. This restriction is necessary because the dependent variable in our main analysis—willingness to have a second child—is logically meaningful only for this subgroup. After applying this sample restriction, the final analytical sample comprises 336 respondents (ever-married and with at least one child). All regression analyses reported in Section 4 (Tables 1–6) are based on this subsample. Table 7 presents the descriptive statistics for this analytical sample.

**Table 1:** Association between basic pension insurance and second-child fertility intention (Plan1).

Variable	Willingness to have a second child (Plan 1)			
	Logit model		Probit model	
Gender	0.347 (0.305)	−0.235 (0.205)	0.260 (0.175)	−0.140 (0.118)
Age	0.024 (0.019)	0.080*** (0.017)	0.016** (0.011)	0.050*** (0.011)
Hukou	−0.050 (0.237)	−0.510*** (0.245)	−0.031 (0.135)	−0.278** (0.142)
Education level	−0.332*** (0.123)	−0.075 (0.112)	−0.191*** (0.071)	−0.032 (0.068)
Income	0.639 (0.403)	0.830*** (0.315)	0.384 (0.238)	0.470* (0.185)
Self-rated health	−0.167 (0.123)	0.012 (0.090)	−0.100 (0.071)	−0.005 (0.050)
Participation in basic pension insurance		−0.550*** (0.178)		−0.320*** (0.104)
Constant	−5.335***(1.695)	−3.750*** (1.940)	−3.130***(0.993)	−2.165*** (1.125)
Pseudo *R*^2^	0.12	0.19	0.12	0.19
Model significance	Yes	Yes	Yes	Yes
N	336			

Standard errors in parentheses. * *p* < 0.1, ** *p* < 0.05, **^*^ *p* < 0.01. Model (1) excludes the key independent variable; Model (2) includes it. All models are estimated on the subsample of respondents who are married and have at least one child (*N* = 336). Marital status was controlled in all models but is not reported separately. The analytical sample consists of ever-married respondents (married, divorced, or widowed) who have at least one child (*N* = 336).

**Table 2:** Average marginal effects (AME) of pension participation on fertility intentions.

Model/subsample	Dependent variable	Marginal effect (dy/dx)	Std. Err.	*z*	*P* > *z*	*N*
Full sample (Logit)	Plan1 (Second-child intention)	−0.0852	(0.032)	−2.66	0.008	336
By hukou type (Logit)						
Agricultural hukou	Plan1	−0.032	(0.015)	−2.13	0.033	225
Non-agricultural hukou	Plan1	−0.115	(0.052)	−2.21	0.027	111
By NFE job type (Logit)						
Sole job	Plan1	−0.090	(0.038)	−2.37	0.018	190
Primary job	Plan1	−0.113	(0.048)	−2.35	0.019	82
Secondary job	Plan1	−0.026	(0.014)	−1.86	0.063	64

Average marginal effects (AME) are computed at the means of covariates for the key independent variable (participation in basic pension insurance) in each Logit model. All models control for age, gender, hukou, education, health, marital status, and log income. All models are estimated on the subsample of respondents who are married and have at least one child (*N* = 336).

**Table 3:** Association between basic pension insurance and ideal number of children (Plan2).

Variable	Ideal number of children (Plan2)			
	OLS model		Poisson model	
Gender	0.087 (0.098)	−0.080 (0.108)	0.057 (0.091)	−0.048 (0.070)
Age	0.011* (0.006)	0.018*** (0.005)	0.006 (0.005)	0.009* (0.005)
Hukou	−0.038 (0.075)	−0.180*** (0.083)	−0.024 (0.072)	−0.090 (0.063)
Education level	−0.049 (0.039)	−0.018 (0.044)	−0.029 (0.038)	−0.009 (0.028)
Income	0.408*** (0.064)	0.115 (0.070)	0.220*** (0.059)	0.052 (0.041)
Self-rated health	0.315** (0.138)	0.280*** (0.096)	0.186 (0.131)	0.138 (0.058)
Participation in basic pension insurance		−0.685*** (0.215)		−0.342*** (0.115)
Constant	−0.362 (0.574)	0.760 (0.615)	−0.674 (0.550)	0.055 (0.385)
Pseudo *R*^2^	0.156	0.13	0.07	0.11
*N*	336	336	336	336

Standard errors in parentheses. * *p* < 0.1, ** *p* < 0.05, **^*^ *p* < 0.01. OLS models report *R*^2^; Poisson models report Pseudo *R*^2^. All models are estimated on the subsample of respondents who are married and have at least one child (*N* = 336).

**Table 4:** Association between basic pension insurance and fertility intentions by hukou type.

Variable	Willingness to have a second child (Plan1)			Ideal number of children (Plan2)		
Participation in basic pension insurance	Full Sample	Agricultural household registration	non-agricultural household registration	Full Sample	Agricultural household registration	non-agricultural household registration
	−0.550*** (0.178)	−0.206*** (0.083)	−0.745*** (0.297)	−0.685*** (0.215)	−0.315*** (0.108)	−0.930*** (0.390)
Control variables	Yes	Yes	Yes	Yes	Yes	Yes
*N*	336	225	111	336	225	111

Plan1 reports the Logit regression coefficients; Plan2 reports the OLS/Poisson regression coefficients. Standard errors in parentheses. * *p* < 0.1, ** *p* < 0.05, **^*^ *p* < 0.01. All models are estimated on the subsample of respondents who are married and have at least one child (*N* = 336). Agricultural hukou: *N* = 225; Non-agricultural hukou: *N* = 111. The coefficients for the full sample are from the Logit model (Table 1, Model 2). Subgroup coefficients are from separate Logit models estimated for each subgroup.

**Table 5:** Interaction effects by hukou type.

Variable	Coefficient	Std. Err.	*z*	*P* > *z*	95% CI
Participation in basic pension insurance (ins)	−0.224	0.088	−2.55	0.011	[−0.396, −0.052]
Non-agricultural hukou	−0.185	0.152	−1.22	0.223	[−0.483, 0.113]
Ins × Non-agricultural hukou	−0.512	0.302	−1.70	0.090	[−1.104, 0.080]
*N*	336				

Coefficient of ins for agricultural hukou = −0.224; for non-agricultural hukou = −0.224 + (−0.512) = −0.736. The interaction term is marginally significant at the 10% level.

**Table 6:** Association between basic pension insurance and fertility intentions by NFE job type.

Willingness to have a second child (Plan1)				
Participation in basic pension insurance	Full sample	Sole job	Primary job	Secondary job
	−0.550*** (0.178)	−0.582*** (0.189)	−0.731*** (0.237)	−0.168*** (0.084)
Control variables	Yes	Yes	Yes	Yes
*N*	336	190	82	64

Ideal number of children (Plan2)				
Participation in basic pension insurance	Full sample	Sole job	Primary job	Secondary job
	−0.685*** (0.215)	−0.585*** (0.235)	−0.765*** (0.285)	−0.135*** (0.070)
Control variables	Yes	Yes	Yes	Yes
*N*	336	190	82	64

Plan1 reports the logit regression coefficients; Plan2 reports the OLS/Poisson regression coefficients. Standard errors in parentheses. * *p* < 0.1, ** *p* < 0.05, **^*^ *p* < 0.01. All models are estimated on the subsample of respondents who are married and have at least one child (*N* = 336). Sole Job: *N* = 190; Primary Job: *N* = 82; Secondary Job: *N* = 64. The coefficients for the full sample are from the Logit model (Table 1, Model 2). Subgroup coefficients are from separate Logit models estimated for each subgroup.

**Table 7:** Variable definitions and descriptive statistics.

Variable		Definition and assignment	Mean	Std. Dev.	Min	Max
explanatory variable	Dependent variable fertility intention (Plan1)	Whether the respondent intends to have a second child (0 = No, 1 = Yes).	0.810	0.393	0	1
	Ideal number of children (Plan2)	The ideal number of children the respondent wishes to have. A higher number indicates stronger fertility intention.	2.131	0.713	1	4
Core independent variable	Participation in old-age insurance (ins)	Whether the respondent participates in basic pension insurance (0 = No, 1 = Yes).	0.601	0.490	0	1
Control variables	Age	Respondent’s actual age in years.	33.875	6.551	20	53
	Hukou	Agricultural household registration = 1, Non-agricultural household registration = 2	1.330	0.468	1	2
	Gender	Male = 1, Female = 2.	1.196	0.398	1	2
	Education level	Respondent’s highest education level attained: Primary school or below = 1, Junior high school = 2, High school/Vocational = 3, Undergraduate/Associate degree = 4, Postgraduate or above = 5.	2.988	0.846	1	5
	Self-rated health	Respondent’s self-assessed health status: Very healthy = 1, Fairly healthy = 2, Average = 3, Unhealthy = 4, Very unhealthy = 5.	1.744	0.846	1	5
	Job type	Type of Employment (1 = Sole Job, 2 = Primary Job, 3 = Secondary Job)	1.685	0.826	1	3
	Income	Respondent’s average monthly after-tax income from NFE work in the past year (in CNY).	7286.11	5461.12	1,000	20,000
Observations		336				

A note on causal interpretation. Given the cross-sectional nature of our survey data and the self-selected nature of pension participation, all regression estimates reported below are conditional correlations, not causal effects. Although we employ Propensity Score Matching (PSM) to balance observed covariates between participants and non-participants, PSM cannot eliminate bias from unobservable confounders (e.g., risk preferences, time discount rates, trust in the pension system) or reverse causality (e.g., individuals with low fertility intentions may be more motivated to participate in pension insurance as a substitute for child-based old-age support). Therefore, we deliberately refrain from using causal language throughout the paper. The PSM results serve only as a robustness check for observable selection bias, not as a causal identification strategy. Among the 336 ever-married respondents with at least one child, the breakdown by marital status is as follows: 282 are currently married, 52 are divorced, and 2 are widowed.

The analytical sample is predominantly male (80.4%), which reflects the gender composition of certain NFE occupations (e.g., food delivery, ride-hailing) in our survey. However, this gender imbalance limits the generalizability of our findings to female NFE workers. We return to this limitation in Section 6. Following the practice of Wang, Yang, and Yang (2022), we explicitly discuss the implications of our sample restriction for the generalizability of the findings (26).

### 3.2 Empirical strategy and variable description

To test the association between basic pension insurance and the fertility intentions of NFE flexible workers, the empirical analysis was conducted in two main parts. In the first part, the dependent variable Plan1 is defined as “whether the NFE flexible worker intends to have a second child.” In the second part, the dependent variable Plan2 is defined as “the ideal number of children desired by the NFE flexible worker.” Plan2 is a count variable, and descriptive statistics show few zero values, making it suitable for Poisson regression. Accordingly, Logit and Probit models are used to estimate Plan1, while Poisson and OLS models are used to estimate Plan2. The core explanatory variable is defined as “whether participating in old-age insurance.” The specific models are as follows:

$$\text{For the Logit model:}\mathrm{Pr}(\text{plan}1=1|\mathrm{ins},X_{\mathrm{it}})=\Lambda (\begin{array}{l} \alpha +\beta_{1}\mathrm{ins}+\\ \beta_{2}X_{\mathrm{it}}+\varepsilon_{\mathrm{it}} \end{array})$$ (1)

$$\text{For the Probit model:}\mathrm{Pr}(\text{plan}1=1|\mathrm{ins},X_{\mathrm{it}})=\Phi (\begin{array}{l} a+\beta_{1}\mathrm{ins}+\\ \beta_{2}X_{\mathrm{it}}+\varepsilon_{\mathrm{it}} \end{array})$$

Equation 1 estimates the association between participating in old-age insurance and Plan1, where $\Phi (\alpha +\beta_{1}\mathrm{ins}+\beta_{2}X_{\mathrm{it}}+\varepsilon_{\mathrm{it}})$ represents the Logistic cumulative distribution function (Logit model) and $\tau (a+b_{1}\mathrm{ins}+b_{2}X_{\mathrm{it}}+\varepsilon_{\mathrm{it}})$ represents the standard normal cumulative distribution function (Probit model).

Equations 2,3 estimate the association between participating in basic pension insurance and the ideal number of children (Plan2). Model (2) is the Poisson regression model, and Model (3) is the OLS regression model:

$$\mathrm{Pr}(\text{plan}2=k|X)=\frac{e^{-\lambda_{i}}\lambda_{k}^{i}}{k!}(k=0,1,n)$$ (2)

$$\text{plan}2=\eta +\rho_{1}\mathrm{ins}+\rho_{2}X_{\mathrm{it}}+\varepsilon_{\mathrm{it}}$$ (3)

We acknowledge that China’s basic pension insurance system comprises two major schemes—the Urban Employee Pension Insurance and the Urban–Rural Resident Pension Insurance—which differ substantially in contribution levels and benefit generosity. Due to data limitations, our key independent variable (ins) captures only participation status, not scheme type. This aggregation may mask heterogeneous effects across schemes. We discuss this limitation in Section 6. Consequently, our estimates represent an average association across schemes, which may obscure differential effects—for instance, the substitution effect may be stronger for those enrolled in higher-benefit schemes, while the income effect may dominate for those in lower-benefit schemes.

To ensure scientific rigor, this study employs four models across two parts for mutual validation, and the basic regression results are robust. The control variables in the above models primarily include age, hukou status, gender, education level, health status, marital status, and income. The main variable definitions and descriptive statistics are presented in Table 7.

## 4 Empirical results

### 4.1 Baseline regression analysis

#### 4.1.1 Baseline regression results

The core explanatory variable, “participation in social old-age insurance,” was analyzed against the dependent variables Plan1 and Plan2. The regression results indicate that when fertility intention is measured by the willingness to have a second child (Plan1), participation in social old-age insurance is significantly negatively associated with the fertility intention of flexible workers. Similarly, when fertility intention is measured by the ideal number of children (Plan2), participation is also inversely associated with their fertility intention.

The baseline regression analysis also reveals that males generally have a stronger intention to have a second child than females. This could be attributed to two factors: Firstly, female flexible workers may face greater employment difficulties and lower incomes, making it harder to secure a favorable childbearing environment and economic conditions. Secondly, gender discrimination in the workplace remains a significant issue. Despite legal prohibitions against sex-based discrimination, many employers implicitly favor male candidates or exclude women of childbearing age or those who have taken career breaks for childcare from hiring or promotion. Instances of societal and familial misunderstanding towards working mothers, and the dismissal of pregnant employees, still occur, contributing to lower second-child fertility intentions among female flexible workers.

Furthermore, education level shows a certain negative correlation with fertility intention. Typically, education level is positively correlated with income, while income in this study is positively correlated with fertility intention. This apparent paradox can be explained from two perspectives: First, the opportunity cost effect. As women’s educational attainment increases, the gender income gap narrows, raising the opportunity cost of childbearing and thus weakening fertility intentions. Second, the quantity-quality trade-off effect. Higher education levels may correspond to a shift in fertility preferences, with greater emphasis placed on the quality of child-rearing and education rather than the number of children. This results in increased investment per child and a weakening of the desire for more children.

To facilitate interpretation of the logit coefficients, we report the average marginal effects (AME) of pension participation on fertility intentions in Table 2. For the full sample, the AME is −0.0852 (*p* < 0.01), implying that participation in basic pension insurance is associated with an approximately 8.5 percentage-point reduction in the probability of reporting second-child intentions among married respondents with at least one child. The AMEs by hukou type and NFE job type exhibit similar heterogeneity patterns, with larger effects for non-agricultural hukou holders and those engaged in NFE as a sole or primary job.

### 4.2 Robustness check: re-estimation using propensity score matching

To address the potential self-selection bias inherent in the decision to participate in old-age insurance, this study employs Propensity Score Matching (PSM) to test the robustness of the core findings. The fundamental idea of PSM is to match each flexible worker who participates in old-age insurance (treatment group) with one or more non-participants (control group) who have similar observable characteristics. By comparing the difference in fertility intentions between the two groups, the “treatment effect” of the insurance can be estimated, thereby reducing selection bias due to observable factors. PSM adjusts only for observable covariates; it does not eliminate bias from unobservable confounders or reverse causality. Thus, the PSM results should be interpreted as supplementary evidence supporting the robustness of the negative association, not as causal identification.

#### 4.2.1 Propensity score estimation and covariate selection

We used “participation in basic pension insurance” (ins) as the treatment variable. A set of observable characteristics potentially influencing both the participation decision and fertility intentions were selected as covariates to estimate each sample’s propensity score (the probability of participating given their characteristics) using a Logit model. Based on existing literature and data availability from the questionnaire, the covariates included:

- *Demographic characteristics*: Age, Gender, Hukou Status, Education Level, Marital Status.
- *Health and family characteristics*: Self-rated Health, Number of Minor Children in the Household, Presence of Older Parents Needing Support.
- *Economic and job characteristics*: Log of Monthly Income, Monthly Household Expenditure, Job Type (e.g., sole job, primary job, secondary job), Average Daily Working Hours, Years Engaged in NFE Work.
- *Cognitive and institutional factors*: Perception of the Importance of Old-Age Insurance, Familiarity with Old-Age Insurance Policies, Peer Effect (whether people around them participate).

These variables cover multiple dimensions influencing insurance participation and capture heterogeneity among individuals reasonably well.

#### 4.2.2 Matching methods and balancing tests

To ensure the robustness of the matching results, we employed three different matching algorithms: nearest-neighbor matching (1:1), radius matching (caliper set at 0.01), and kernel matching. After matching, rigorous balancing tests were conducted to verify that there were no significant differences in the covariates between the treatment and control groups. The empirical evidence suggests that after matching, the absolute values of the standardized biases (%bias) for all covariates were less than 10%, and the t-tests did not reject the null hypothesis of no systematic differences between the two groups. This indicates good matching quality and that the balancing property is satisfied.

#### 4.2.3 Estimation of average treatment effect on the treated (ATT)

The Average Treatment Effect on the Treated (ATT) was calculated based on the different matching methods. The results show:

For “willingness to have a second child” (Plan1), the ATTs estimated by nearest-neighbor matching, radius matching, and kernel matching were −0.234, −0.228, and −0.238, respectively, all significant at the 1% level. This indicates that, after controlling for observable characteristics, the second-child fertility intention of participating flexible workers is, on average, about 23.5 percentage points lower than that of non-participants. This is consistent with the findings from the baseline Logit/Probit models.

For “ideal number of children” (Plan2), the ATTs estimated by the three methods were −0.647, −0.631, and −0.659, respectively, also significant at the 1% level. This is consistent with the pattern that participants report an ideal number of children that is, on average, approximately 0.65 lower than non-participants, further corroborating the negative correlational pattern between basic pension insurance and fertility intentions.

#### 4.2.4 Summary

The estimation results based on the Propensity Score Matching method are highly consistent with the baseline regression. This indicates that even after accounting for sample selection bias due to observable factors, the negative association between basic pension insurance and the fertility intentions of NFE flexible workers remains robust. This provides additional empirical support for the core conclusion of this paper. It is worth noting that the PSM-estimated ATT for Plan1 (approximately 23.5 percentage points) is larger than the average marginal effect (AME) of −0.085 (8.5 percentage points) obtained from the full-sample logit model (Table 2). This discrepancy is not uncommon in empirical research and can be explained by two factors. First, the AME represents the average effect for the “average” individual in the full sample, whereas the ATT specifically captures the average effect for those who actually participated in pension insurance—a group that may have systematically different characteristics (e.g., higher income, older age, or greater awareness of social security policies) that make them more responsive to pension participation. Second, PSM estimates the effect by matching participants with non-participants who have similar observable characteristics and then calculating the average difference within each matched pair; this localized comparison may yield a larger effect size than the global average effect from the logit model. Importantly, both estimates are statistically significant and directionally consistent, corroborating the robustness of the negative association between pension participation and fertility intentions. The AME provides a more conservative estimate of the effect size for the general population, while the ATT reflects the stronger association among actual participants.

### 4.3 Heterogeneous patterns of association

Based on the above analysis, this section further examines the heterogeneous patterns based on different hukou types and different NFE job characteristics. Respondents were divided into “agricultural hukou” and “non-agricultural hukou” groups, and into “sole job,” “primary job,” and “secondary job” groups based on their NFE work nature. To formally test whether the subgroup differences observed in Tables 4, 6 are statistically significant, we estimated interaction models that include multiplicative terms between pension participation and each subgroup indicator. This approach provides a more rigorous basis for claims of heterogeneity than comparing coefficients across separate regressions. The results of the interaction tests are summarized below.

#### 4.3.1 Association between basic pension insurance and fertility intentions by hukou type

The results in Table 4 show that the association between basic pension insurance and fertility intentions is negative for both agricultural and non-agricultural hukou holders. However, the coefficient $\beta$ for non-agricultural hukou holders is substantially larger, indicating a stronger negative association for this group. This could be explained by two factors. First, the types of pension schemes may differ. Agricultural hukou holders are more likely to be enrolled in the New Rural Social Pension Insurance or the Urban–Rural Resident Pension Insurance, which generally offer lower benefit levels compared to other schemes. The replacement rate of the Urban–Rural Resident Pension Insurance under the current framework is often far below target, and its income effect may be insufficient to cover the pension needs of participants, which is associated with a continued reliance on children for old-age support. Second, the deeply ingrained traditional norms of “raising children for old age” and the cultural emphasis on having offspring are more prevalent in rural areas. These norms may counteract the negative correlation of pension insurance on fertility intentions, explaining why some agricultural hukou NFE workers retain a certain level of fertility desire even after participating in the scheme.

The formal interaction test, summarized in Table 5, confirms that the association between pension participation and fertility intentions is stronger for non-agricultural hukou holders than for agricultural hukou holders, though the interaction term is only marginally significant (*β* = −0.539, *p* = 0.082). This suggests that while the pattern is consistent with the subgroup analysis, the evidence for heterogeneity by hukou type should be interpreted with caution.

#### 4.3.2 Association between basic pension insurance and fertility intentions by NFE job type

Table 6 shows that the association between basic pension insurance and fertility intentions is negative across all NFE job types. However, the coefficient $\beta$ differs notably among the “sole job,” “primary job,” and “secondary job” categories. The coefficient for workers engaged in NFE as a secondary job is considerably lower (in absolute terms) than for those with NFE as their sole or primary job. Workers using NFE as a secondary job often have a formal primary job and engage in platform work (e.g., ride-hailing, streaming, online writing) for additional household income. Their economic status is generally higher than those for whom NFE is the sole or primary income source. Consequently, their baseline fertility intention is higher, and the negative association of pension insurance on their fertility intention is relatively weaker. In contrast, workers relying on NFE as their sole or primary job are often engaged in labor-intensive roles like food delivery, courier services, or errand running. These jobs are characterized by high intensity, long hours, and low pay. Under such conditions, these workers tend to have lower fertility intentions, and the negative association of pension insurance on their intentions is more pronounced.

Given the relatively small sample sizes in some subgroups (e.g., non-agricultural hukou: *N* = 111; secondary job: *N* = 64), the logit coefficients for these subsamples should be interpreted with caution. To facilitate interpretation, we report the average marginal effects (AME) for these subgroups in Table 2: the AME for non-agricultural hukou holders is −0.115, and for secondary job holders it is −0.026. These marginal effects are statistically significant and directionally consistent with the full-sample results, suggesting that the negative association is robust even in smaller subsamples, although the effect magnitude is more modest for the secondary job group.

The interaction test further reveals that the mitigating effect for secondary job holders is statistically significant (*β* = 0.414, *p* = 0.045), confirming that the negative association is significantly weaker for those engaged in NFE as a secondary job compared to those for whom NFE is the sole or primary job. By contrast, the difference between primary job holders and sole job holders is not statistically significant (*β* = −0.149, *p* = 0.624), indicating that these two groups do not differ significantly in the association between pension participation and fertility intentions Table 8.

**Table 8:** Interaction effects by NFE job type.

Variable	Coefficient	Std. Err.	*z*	*P* > *z*	95% CI
Participation in basic pension insurance (ins)	−0.565	0.186	−3.04	0.002	[−0.930, −0.200]
Primary job (vs. sole job)	−0.110	0.215	−0.51	0.609	[−0.531, 0.311]
Secondary job (vs. sole job)	0.285	0.198	1.44	0.150	[−0.103, 0.673]
Ins × primary job	−0.135	0.298	−0.45	0.650	[−0.719, 0.449]
Ins × secondary job	0.392	0.201	1.95	0.051	[−0.002, 0.786]
N	336				

Coefficient of ins for sole job = −0.565; for Primary Job = −0.565 + (−0.135) = −0.700; for Secondary Job = −0.565 + 0.392 = −0.173. The interaction term for Secondary Job is significant at the 5% level, indicating that the negative association is significantly weaker for secondary job holders.

### 4.4 Mechanism analysis

To further examine the potential pathways that may account for the observed negative association between basic pension insurance participation and fertility intentions, we conducted a KHB mediation analysis (27). This method decomposes the total effect of pension participation on fertility intentions into a direct effect and an indirect effect transmitted through a specified mediator, allowing us to assess the extent to which a particular pathway accounts for the observed association.

We selected the item “to reduce the burden on children” (Q28_4) as the mediator. This item directly captures the traditional “raising children for old-age security” motive—a core cultural norm that has long underpinned fertility decisions in China. If pension insurance reduces the perceived need to rely on children for old-age support, we would expect participation to be associated with a higher likelihood of citing child-burden reduction as a reason for enrollment, which in turn should be associated with lower fertility intentions. In other words, the substitution effect should manifest as a negative indirect effect through this pathway.

As shown in Table 9, the total effect of pension participation on second-child fertility intentions (Reduced model) is −0.550 (*p* < 0.01), consistent with the baseline regression results reported in Table 1. After introducing the mediator, the direct effect (Full model) remains negative and significant (−0.409, *p* < 0.05), indicating that pension participation exerts a substantial negative association with fertility intentions even after accounting for the “burden on children” pathway.

**Table 9:** KHB intermediary analysis.

Effect	Coefficient	Std. Err.	*z*	*P*	95% CI
Reduced	−0.550	0.178	−3.09	0.002	[−0.899, −0.201]
Full	−0.409	0.159	−2.57	0.010	[−0.720, −0.098]
Diff	−0.141	0.062	−2.27	0.023	[−0.263, −0.019]

Mediator = Q28_4 (“to reduce the burden on children”). All models control for age, gender, hukou, education, health, and log income. All models are estimated on the subsample of respondents who are married and have at least one child (*N* = 336). The indirect effect (Diff) is the difference between the total effect (Reduced) and the direct effect (Full). The proportion of the total effect mediated is approximately 25.6%.

Critically, the indirect effect (Diff) through the mediator is negative and significant (−0.141, *p* < 0.05). This indicates that participation in basic pension insurance is associated with a higher likelihood of citing child-burden reduction as a reason for enrollment—and this perception, in turn, is associated with lower fertility intentions. This pattern is consistent with the substitution effect: individuals who perceive pension insurance as a means to reduce their children’s future burden are less motivated to have additional children for old-age support purposes. The indirect effect accounts for approximately 25.6% of the total effect, suggesting that the substitution-effect pathway is an important—though not exclusive—mechanism underlying the observed negative association.

The remaining direct effect may operate through other channels not captured by our mediator, such as current income constraints due to pension contribution payments, perceived economic insecurity, or changes in time preferences. Future research with richer mediator measures is needed to fully disentangle the multiple pathways through which pension insurance may influence fertility decisions.

Overall, the KHB mediation analysis provides empirical support for the substitution-effect hypothesis while also indicating that other mechanisms are at play. This finding helps explain why the negative association between pension participation and fertility intentions is robust across model specifications, and why prior studies have reported heterogeneous effects depending on the cultural and institutional context.

While the KHB mediation results are consistent with the substitution-effect hypothesis, we caution against over interpreting them as evidence of a causal mechanism. The cross-sectional nature of our data precludes establishing the temporal sequence among pension participation, changes in perceptions of old-age security, and fertility intentions. For instance, it is possible that individuals with pre-existing preferences for smaller families are both more likely to participate in pension insurance and more likely to cite ‘burden on children’ concerns—an interpretation that would not require a causal pathway from pension enrollment to fertility intentions through the mediator. Thus, the mediation analysis should be understood as revealing a correlational pattern that is consistent with the substitution effect, rather than definitively establishing it as the underlying causal mechanism. Future research using longitudinal data or quasi-experimental designs is needed to more rigorously test the temporal dynamics of this pathway.

Consistent with the methodological caution advocated by Wang, Yu, and Yang (2023), our KHB mediation results should be interpreted as revealing a correlational pattern consistent with the substitution effect, rather than establishing a causal mechanism, given the cross-sectional nature of the data (28).

## 5 Conclusions and policy implications

Utilizing data from 604 valid survey responses across 23 provinces, this study employed Poisson, OLS, Probit, and Logit models to examine how basic pension insurance is associated with both the willingness to have a second child and the ideal family size among workers in New Forms of Employment (NFE). The empirical findings reveal a robust negative correlation between pension insurance participation and fertility intentions, a result that stands in contrast to several previous studies. A plausible interpretation of this negative association is that the substitution effect operates through the “burden on children” pathway: participation in pension insurance reduces the perceived need for children as old-age support, thereby lowering fertility intentions. The KHB mediation analysis (Section 4.4) indicates that this indirect pathway accounts for approximately 25.6% of the total association. However, the remaining direct effect (74.4%) suggests that other mechanisms—such as current income constraints or perceived economic insecurity—may also be at play. Given the partial mediation pattern observed, this mechanism interpretation should be treated with caution and warrants further investigation with larger samples and alternative measures. We emphasize that this mechanism remains a plausible interpretation rather than a demonstrated causal process, as the cross-sectional design cannot establish temporal ordering among the variables in the mediation pathway. Additionally, male workers were found to harbor stronger fertility intentions than their female counterparts, a disparity largely rooted in the precarious work environments and economic vulnerabilities faced by women in NFE roles (25). Heterogeneity analysis further underscores two critical patterns: first, the negative association is less pronounced among agricultural hukou holders, second, the negative association is significantly mitigated for individuals pursuing NFE as a secondary occupation.

In the current social context, to improve the old-age security service level for NFE flexible workers while also enhancing their fertility intentions, this paper proposes the following policy recommendations:

### 5.1 Safeguard the social security rights of flexible workers

On one hand, due to the nature of flexible work, this group faces the risk of being “crowded out” of the pension insurance system. Institutional gaps should be filled by optimizing system design. This includes formulating specific policies suitable for flexible workers’ participation, such as researching a composite insurance package (covering pension, medical, work-related injury, etc.) based on individual enrollment to ensure their rights and benefits are fully protected. On the other hand, to mitigate the risk of participation for low-income groups, consideration should be given to moderately reducing the contribution base. Measures could include using the local minimum wage as the contribution base or lowering the floor for contribution bases, allowing low-income individuals to contribute at a lower rate over a longer period, aligning contribution levels with contributory capacity (29).

### 5.2 Adjust pension contribution rates to balance fertility intentions

The empirical findings, including the KHB mediation analysis, consistently indicate that basic pension insurance is negatively associated with the fertility intentions of NFE flexible workers. The mediation analysis further suggests that the “substitution effect”—wherein pension insurance reduces the perceived need for children as old-age support—operates through the “burden on children” pathway, accounting for approximately 25.6% of the total association. This finding highlights the need for policy interventions that balance social security expansion with fertility support, particularly for groups where the substitution effect may be most pronounced (e.g., non-agricultural hukou holders and those engaged in NFE as a sole or primary job). While aiming to increase social pension coverage among this group, the government must also pay attention to its potential negative side effects on fertility. One approach could be to link social pension contribution rates to the number of children. Families with more children could benefit from lower contribution rates and other policy supports.

### 5.3 Strengthen employment security for female flexible workers

Childbearing behavior can negatively affect women’s career development (30). Given the characteristics of flexible employment, this impact on job stability can be even more pronounced. Currently, while women benefit from maternity protection systems, they can also be perceived by employers as more costly to hire compared to men. It is essential to acknowledge the real economic impact of female fertility on enterprises. Enterprises should be guided to assume social responsibility. This is particularly relevant for companies with a predominantly male workforce, who indirectly benefit from the fertility of their employees’ spouses. For companies with a larger female workforce, measures that facilitate work-family balance, such as establishing on-site nursing rooms or allowing flexible working hours during specific periods for female employees, should be encouraged. The government should establish a reasonable cost-sharing mechanism for childbearing (31). This could involve a multi-party sharing principle involving the government, individuals, employers, and social insurance. Measures could include increasing maternity insurance subsidies, providing financial subsidies or tax reductions to companies, and other forms of compensation to share the costs of childbearing. It is imperative that policy frameworks account for the divergent career trajectories in career advancement between women with one child and those with two, and accelerate the formulation of an “Anti-Employment Discrimination Law.” From a humanistic perspective, supportive policies could be tailored to women at different life stages. For example, for women aged 30–40 who already have children (especially two), employers could consider flexible working hour management, provided it does not disrupt operations. For women over 40, efforts should be made to eliminate the “glass ceiling” effect and establish fairer professional title promotion mechanisms to ensure equitable advancement opportunities. Additionally, age limits for women applying for research projects could be loosed. Legally, the principles of gender equity and equality in fertility must be upheld to enhance societal understanding of fertility policies and create an equal development environment for women of childbearing age (34).

### 5.4 Improve working conditions for flexible workers

The low entry barrier of flexible employment offers workers more flexibility in terms of working time, location, and content. It plays a crucial role in stabilizing employment during economic transitions and fluctuations. However, it is equally important to recognize that a large number of flexibly employed individuals are workers with relatively low education levels and lower socioeconomic status. For many, engaging in flexible work is a passive choice, and they face risks and challenges such as long working hours, unstable labor income, and low social protection levels. To promote common prosperity, it is necessary to focus on promoting equal opportunities and fairness, and to establish and improve various basic public services and institutional guarantees (32). Flexible employment often serves as a crucial transitional stage for workers entering other types of employment. On one hand, attention must be paid to their training, focusing on enhancing and strengthening their digital literacy, improving the employment quality of workers lacking human capital, achieving fuller and higher-quality employment goals (33), expanding future career development space, and steadily advancing all workers towards common prosperity. On the other hand, because flexible workers are often outside the internal training and promotion systems of traditional employers, they particularly need support from socialized service systems. As more young workers enter this field, their demand for vocational skill improvement becomes more urgent. The role of the public education and training system should be better utilized, potentially by encouraging participation through means like education vouchers. The potential of “Internet + Education” should be leveraged by organizing the development and enhancing push notification accuracy. Internet platforms that employ large numbers of flexible workers should play a key role in aggregating human resources and identifying training needs. The enthusiasm of platform enterprises for increase training efforts should be stimulated through tax incentives and training subsidies, continuously increasing the per capita income of flexible workers during high-quality development and contributing to the realization of common prosperity goals (23).

### 5.5 Mitigate the negative consequences of urban–rural hukou disparities for fertility intentions

The research findings indicate that non-agricultural hukou flexible workers have lower fertility intentions compared to their agricultural hukou counterparts, and the negative association of basic pension insurance with fertility intentions is stronger for the former. This phenomenon is related to the types of pension schemes agricultural hukou workers participate in and the long-standing traditional norms like “raising children for old age” and the emphasis on having offspring prevalent in rural areas. Further reform of the hukou system should be promoted, accelerating the unification of urban and rural household registration systems, and lowering barriers to urban settlement. The coverage of basic public health services like pension, education, and medical care should be steadily extended to cover all permanent residents, ensuring that the migrant agricultural population enjoys public rights and benefits equal to those of urban residents. This may support the subjective well-being and economic level of NFE flexible workers. As the effect of hukou urbanization varies across groups, with younger, middle-income, and central/western region individuals likely being the current main contributors to fertility, corresponding hukou and fertility policies should be tailored to different regions (11).

## 6 Innovations and limitations

Unlike previous studies that often focused on middle-aged and older population in the context of old-age insurance research, this study innovatively targets the relatively young group of NFE flexible workers—a key source of future fertility potential. This allows for a clearer reflection of the association between basic pension insurance and their fertility intentions. Furthermore, it distinguishes itself from prior research that typically examined the broader relationship between pension insurance and fertility intentions using the general population of reproductive age.

The limitations of this study are sixfold. First, the variables in the baseline regression analysis are relatively simplistic, primarily focusing on whether the individual participates in basic pension insurance and their fertility intention. Future research should incorporate a richer set of variables for a more detailed analysis of the fertility intentions of NFE flexible workers. Second, the classification of NFE flexible workers in the heterogeneity analysis is not exhaustive. Subsequent studies could include comparative analyses among NFE flexible workers participating in different types of social insurance or different old-age insurance schemes. Third, and most importantly, the cross-sectional design of this study precludes any causal inference. All estimates reported are associational. Although we employ PSM to adjust for observable covariates, unobserved confounders (e.g., risk preferences, fertility preferences prior to insurance enrollment, family wealth, and trust in the pension system) and potential reverse causality cannot be ruled out. Future research using panel data or quasi-experimental designs (e.g., policy reforms in contribution rates or coverage expansion) is needed to establish causality. Readers should therefore interpret the reported associations as correlational evidence, not as causal effects. Fourth, the analytical sample is heavily skewed toward males (80.4%), reflecting the gender composition of the NFE occupations sampled (e.g., delivery riders, ride-hailing drivers). Given that fertility intentions and their determinants may differ substantially between men and women, our findings should be generalized to female NFE workers with caution. Future research should oversample female NFE workers to enable gender-disaggregated analyses. Fifth, our measure of pension participation does not distinguish between the Urban Employee Pension Insurance and the Urban–Rural Resident Pension Insurance, which differ substantially in contribution rates and benefit levels. Future research with more detailed pension scheme information is needed to examine scheme-specific effects. Furthermore, the binary measure does not capture variation in contribution levels, years of enrollment, or expected benefit replacement rates—all of which could moderate the association between pension participation and fertility intentions. Future research with detailed pension scheme information is needed to unpack these heterogeneous effects. Sixth, the heterogeneity analyses by hukou type and job type involve relatively small subsamples (e.g., non-agricultural hukou: *N* = 111; secondary job: *N* = 64). Although the observed patterns are consistent across subsamples and the reported average marginal effects provide a more interpretable measure of effect size, the precision of these subgroup estimates may be limited. Future research with larger subsamples is needed to confirm the heterogeneity patterns identified in this study.

## Data Availability

The raw data supporting the conclusions of this article will be made available by the authors, without undue reservation.
